# Effects of lemborexant on sleep quality and its association with morning alertness: Post hoc analysis of two phase 3 trials

**DOI:** 10.1016/j.sleepx.2026.100183

**Published:** 2026-03-05

**Authors:** Masahiro Suzuki, Takuya Yoshiike, Atul Khullar, Yuki Kogo, Kanako Inabe, Michinori Koebis, Margaret Moline, Jocelyn Y. Cheng, Dinesh Kumar, Kate Pinner, Kenichi Kuriyama

**Affiliations:** a Department of Psychiatry, Nihon University School of Medicine, 30-1 Oyaguchi-Kamicho, Itabashi-ku, Tokyo, 173-8610, Japan; b Department of Sleep-Wake Disorders, National Institute of Mental Health, National Center of Neurology and Psychiatry, 4-1-1, Ogawa-Higashi, Kodaira, Tokyo, 187-8553, Japan; c University of Alberta, Northern Alberta Sleep Clinic, Alberta, Edmonton, AB, T5N 3Y6, Canada; d Eisai Co., Ltd., 4-6-10 Koishikawa, Bunkyo-ku, Tokyo, 112-8088, Japan; e Eisai Inc., 200 Metro Blvd, Nutley, NJ, 07110, USA; f Eisai Ltd., Hatfield, AL10 9SN, UK

**Keywords:** Subjective sleep quality, Orexin receptor antagonist, Lemborexant, Morning alertness

## Abstract

**Study objectives:**

This analysis was conducted to determine whether lemborexant (LEM) treatment affects ratings of patient-reported sleep quality and to identify subjective and objective parameters associated with improved subjective sleep quality.

**Methods:**

This was a post hoc analysis of two global phase 3 trials (Study 303: 12-month, randomized, double-blind, placebo-controlled trial; Study 304: 1-month, randomized, double-blind, placebo-controlled, active-comparator trial) of LEM for patients with insomnia. A 9-point Likert scale (higher numbers indicating better quality) was used to assess subjective sleep quality (sQual). Subjective and objective sleep parameters were assessed by electronic sleep diaries and polysomnography, respectively. Spearman's rank correlation analysis and stepwise regression analysis were performed to explore the associations between sleep parameters and sQual.

**Results:**

The analyses included 949 patients from Study 303 and 743 patients from Study 304. LEM showed significantly larger increases from baseline in sQual than placebo in both studies (least square mean change: placebo 0.89, LEM5 1.17 (p < 0.05), LEM10 1.21 (p < 0.05) at Month 6 in Study 303, and placebo 0.93, LEM5 1.46 (p < 0.001), LEM10 1.35 (p < 0.05) at Month 1 in Study 304). Of subjective sleep parameters, changes in morning alertness showed the strongest association with changes in sQual. The changes in subjective total sleep time and wake after sleep onset were moderately correlated with sQual positively and negatively, respectively. These findings were further confirmed using stepwise regression analyses.

**Conclusions:**

LEM improved ratings of sQual more than placebo. To improve self-reported sleep quality, lemborexant treatment should focus on improving morning alertness.

**Clinical trial registration:**

Study 303 (ClinicalTrials.gov NCT02952820): Long-term Study of Lemborexant in Insomnia Disorder (SUNRISE 2). https://clinicaltrials.gov/study/NCT02952820, Study 304 (ClinicalTrials.gov NCT02783729): Study of the Efficacy and Safety of Lemborexant in Subjects 55 Years and Older with Insomnia Disorder (SUNRISE 1). https://clinicaltrials.gov/study/NCT02783729.

## Highlights

• LEM significantly improved subjective sleep quality ratings compared to placebo.
• Changes in morning alertness showed the strongest association with changes in sQual.
• Changes in sQual ratings correlated moderately with wake after sleep onset.
• Insomnia treatment should enhance alertness and focus on improving sleep continuity.

## Graphical abstract

**Figure:**
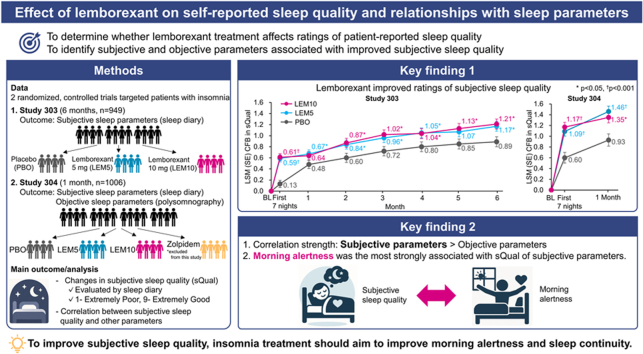

## 1 Introduction

The importance of maintaining good health status through appropriate sleep duration has been highlighted because both short and long sleep durations are associated with various adverse clinical outcomes including all-cause mortality, cardiovascular disease, and diabetes mellitus [[1], [2], [3], [4], [5], [6], [7]]. In addition, the importance of maintaining sleep with good quality for health has also been emphasized. Numerous studies have suggested that poor sleep quality is associated with worse clinical outcomes in various diseases and conditions [[8], [9], [10], [11]].

The definition of “Sleep quality” has not been established and is often multi-dimensionally assessed by using self-reported questionnaires such as the Pittsburgh Sleep Quality Index (PSQI). It has also been tried to figure out with actigraphy and polysomnography (PSG). Objective sleep quality is often defined by total sleep time, sleep efficiency, or several elements of sleep architecture; however, these parameters are not well correlated with ratings of subjective sleep quality, suggesting objective and subjective sleep quality ratings measure different aspects of sleep quality [12,13].

Several studies have demonstrated a relationship between subjective sleep quality and health-related outcomes [9,[14], [15], [16]], including patients with insomnia [[17], [18], [19]]. Therefore, subjective sleep quality can be a crucial index of treatment outcomes for insomnia. Cognitive behavioral therapy for insomnia has been shown to improve self-reported sleep quality assessed by the PSQI [20]. Clinical trials have also demonstrated that several sleep medications improve subjective sleep quality [[21], [22], [23], [24]]. Thus, identifying the sleep parameters that affect ratings of subjective sleep quality may be more useful for treatment strategies to improve subsequent health-related outcomes. Nevertheless, few reports have examined the relationships between subjective sleep quality and specific sleep parameters, especially in the treatment of insomnia. A previous study showed that sleep maintenance and feeling refreshed after sleep predict subjective sleep quality in patients with insomnia [25]; therefore, improving sleep continuity may be a good target for enhancing subjective sleep quality. However, long-acting hypnotics, which are more effective in improving sleep continuity than short-acting hypnotics, are more likely to have daytime carry-over effects leading to poor morning alertness, daytime function, and quality of life (QOL) [26]. Therefore, insomnia medications that enhance sleep maintenance and are associated with good alertness the following morning are needed, since such medication would simultaneously improve subjective sleep quality.

Lemborexant (LEM) is a dual orexin receptor antagonist whose efficacy for treating patients with insomnia was demonstrated in two Phase 3 studies [[27], [28], [29]]. Study E2006-G000-303 (Study 303) showed LEM's efficacy compared with placebo up to 6 months by improving subjective sleep onset and sleep maintenance problems (Table S1) [28,29]. Study E2006-G000-304 (Study 304) demonstrated that LEM led to greater and significant changes from baseline in subjective and objective sleep parameters, including wake after sleep onset (WASO), compared with placebo and zolpidem extended-release (ZOL-ER) during a 1-month treatment period (Table S1) [27]. In addition, LEM showed limited morning residual effects across these studies [[27], [28], [29], [30], [31]]. Since LEM reduced WASO without significant residual effects, the ratings of subjective sleep quality were expected to improve as well.

The objectives of this post hoc analysis were to investigate more thoroughly the effect of LEM on ratings of subjective sleep quality, and to identify changes in parameters associated with changes in subjective sleep quality during LEM treatment.

## 2 Methods

### 2.1 Study design

This was a post hoc analysis of a randomized, double-blind, placebo-controlled, global phase 3 Study 303 (E2006-G000-303, SUNRISE 2, Clinical Registry No. NCT02952820) and a randomized, double-blind, placebo- and active comparator-controlled, global phase 3 Study 304 (Study E2006-G000-304, SUNRISE 1, Clinical Registry No. NCT02783729) [28,30,32]. These studies were conducted in compliance with government notices and guidelines, with the necessary ethical approvals and the informed consent of all patients. The manuscript was reported according to the Consolidated Standards of Reporting Trials (CONSORT) guideline.

Study 303 included patients with Diagnostic & Statistical Manual of Mental Disorders (DSM)-5-diagnosed insomnia disorder who were ≥18 years old (range 18 to 88 years) [28]. Patients with sleep disorders other than insomnia were excluded. Patients with medical and psychiatric conditions were permitted if their condition was stable, adequately controlled, and not treated with a prohibited medication. About 90% of patients had a history of at least one medical condition (Table S2). Study 303 was a 12-month study divided into two treatment periods. A placebo run-in period of the randomized phase was set (as shown in the previous report), and the data during the placebo run-in period were collected and used as baseline data [28]. During treatment period 1 over the first 6 months, patients were assigned to placebo, LEM 5 mg (LEM5), or LEM 10 mg (LEM10) at a 1:1:1 ratio. Randomization was stratified by country and age group (<65 years and ≥65 years). During treatment period 2 over the last 6 months, patients re-randomized to placebo in treatment period 1 were reassigned to LEM5 or LEM10 at a 1:1 ratio, and patients assigned to LEM continued on their original dose. Re-randomization was also stratified by country and age group (<65 years and ≥65 years) in period 2. This post hoc analysis used only the data of period 1.

Study 304 was a one-month study that included women ≥55 years old and men ≥65 years old with DSM-5-diagnosed insomnia disorder [27]. Patients with medical and psychiatric conditions were permitted if their condition was stable, adequately controlled, and not treated with a prohibited medication. More than 90% of patients had a history of at least one medical condition (Table S3). A placebo run-in period of the randomized phase was set (as shown in the previous report), and the data during the placebo run-in period were collected and used as baseline data [33]. The patients were randomized to placebo, LEM5, LEM10, or ZOL-ER at a 4:5:5:5 ratio. Randomization was stratified by country and age group (55–64 years and ≥65 years). To focus on the effect of LEM, ZOL-ER data are not included in the present analyses, since agents with different mechanisms of action may exert distinct effects on sleep parameters, potentially confounding the interpretation of outcomes and detracting from the study's primary focus.

### 2.2 Outcomes

#### 2.2.1 Sleep diary outcomes

Sleep quality endpoints, including subjective sleep quality (sQual) and subjective morning alertness, were analyzed using data from electronic sleep diaries completed daily by each patient within 1 h of waking; sQual was assessed by the question “How would you rate the quality of your sleep last night?” on a 9-point scale, which ranged from 1, representing “Extremely Poor,” to 9, representing “Extremely Good.” Subjective morning alertness was assessed by the question “How sleepy/alert do you feel this morning?” on a 9-point scale, which ranged from 1, representing “Extremely Sleepy,” to 9 representing “Extremely Alert”; 5 was neutral on both scales. These scales are described in detail in Table 1.

**Table 1:** The scale of subjective sleep quality and morning alertness.

Subjective sleep quality	Subjective morning alertness
How would you rate the quality of your sleep last night?	How sleepy/alert do you feel this morning?
1 Extremely Poor	1 Extremely Sleepy
2 Very Poor	2 Very Sleepy
3 Moderately Poor	3 Moderately Sleepy
4 Slightly Poor	4 Slightly Sleepy
5 Neither Good nor Poor	5 Neither Alert nor Sleepy
6 Slightly Good	6 Slightly Alert
7 Moderately Good	7 Moderately Alert
8 Very Good	8 Very Alert
9 Extremely Good	9 Extremely Alert

Sleep onset and sleep maintenance endpoints included: subjective sleep-onset latency (sSOL), the estimated time from attempt to sleep until sleep onset; subjective wake after sleep onset (sWASO), subject-estimated sum of time of being awake during the night after initial sleep onset until the time of getting out of bed in the morning; subjective total sleep time (sTST), which was derived from the minutes spent asleep during the time in bed; and calculated subjective sleep efficiency (sSE), total time spent asleep divided by time in bed, which was calculated using sleep diary entries.

For all sleep diary endpoints, the reported values for the above outcomes were the means of the 7-night records. Baseline data were calculated from the data during the last 7 nights of the placebo run-in period, and the data of the 7 nights before a given study visit were used at months 1–6 in both studies.

#### 2.2.2 Insomnia Severity Index (ISI)

This self-completed questionnaire consists of 7 items. Each item is scored on a 5-point scale of 0 (none) to 4 (very severe). The classification of the ISI total score is as follows: absence of insomnia (0–7), sub-threshold insomnia (8–14), moderate insomnia (15–21), and severe insomnia (22–28) [34]. The sum of items 4 to 7 was used as the score for ISI-based daytime function. The questionnaire was given at baseline and Day 30 of the treatment period in Study 304 and at baseline and Months 1, 3, and 6 in Study 303.

#### 2.2.3 Fatigue Severity Scale (FSS)

This self-completed scale to evaluate the level of agreement with 9 statements is scored on a 7-point scale from 1 (disagree) to 7 (agree). Higher total scores indicate a greater degree of fatigue. The scale was completed at baseline and Day 30 of the treatment period in Study 304 and at baseline and Months 1, 3, and 6 in Study 303.

#### 2.2.4 Objective sleep parameters

In Study 304, patients underwent PSG at baseline, Day 1/2, and Day 29/30, and the two-day means were calculated. The following measures were calculated: latency to persistent sleep (LPS), defined as minutes from lights off to the first epoch of 20 consecutive 30-s epochs of non-wakefulness; WASO, minutes of being awake from LPS until lights on; and sleep efficiency (SE), proportion of time spent asleep per time in bed, calculated as total sleep time (TST)/interval from lights off until lights on [standardized at 8 h].

### 2.3 Statistical analysis

The full analysis set (FAS, i.e., patients who received the study drug at least once) was used for the analyses.

Changes in sQual were analyzed with a mixed-model repeated measures (MMRM) model with factors of region, age group (<65 years, ≥65 years), treatment group, time point, and treatment group-time point interactions as fixed effects and baseline sleep quality as a covariate. Missing values were imputed with a missing at random approach. Subgroup analysis was performed based on sex and age (<65 years and ≥65 years).

A responder analysis was performed to calculate the proportion of patients who had a score >5 (responder) or ≤5 (“neither good nor bad”) of the average of the 7 nights of the last week of months 1 and 6 of treatment for each subgroup: those with a baseline value ≤ 4 (the median) and those >4.

Spearman's rank correlation coefficients were calculated between change from baseline in ratings of sQual and changes from baseline in each subjective sleep parameter, subjective morning alertness, total score of ISI items 4 to 7, and FSS, at 1 and 6 months in Study 303 and at 1 month in Study 304. Correlation coefficients were calculated between change from baseline in ratings of sQual and changes from baseline in each objective sleep parameter at one month in Study 304. Correlation strengths were classified as follows: 0.00*–*0.10, negligible correlation; 0.10*–*0.39, weak correlation; 0.40*–*0.69, moderate correlation; 0.70*–*0.89, strong correlation; and 0.90*–*1.00, very strong correlation [35].

Stepwise regression analysis with backward elimination was performed to examine how subjective sleep parameters affect sQual according to the following equation.

$$\text{sQual}=X_{1}*\text{sSOL}+X_{2}*\text{sWASO}+X_{3}*\text{sTST}+X_{4}*\text{morning}\;\text{alertness}+E\;\left(\text{residual}\right)$$

The analysis was performed in Study 303 at 1 and 6 months and in Study 304 at 1 month.

Statistical analyses were performed using SAS version 9.4 (SAS Institute).

## 3 Results

### 3.1 Patients’ demographic characteristics

The analyses included 949 patients from Study 303 (placebo: 318, LEM5: 316, LEM10: 315) and 743 patients from Study 304 (placebo: 208, LEM5: 266, LEM10: 269). The mean ages of the two patient groups were as follows: in Study 303, placebo 54.5 years, LEM5 54.2 years, and LEM10 54.8 years; and in Study 304, placebo 63.4 years, LEM5 63.7 years, and LEM10 64.2 years. The corresponding proportions of female patients were 67.9%, 66.1%, and 70.5% in Study 303, and 88.5%, 86.1%, and 85.5% in Study 304 for placebo, LEM5, and LEM10, respectively (Table 2). The mean sQual rating in each study at baseline was about 4 points. The patients’ demographic characteristics did not differ notably among the 3 groups within a study [27,28].

**Table 2:** Patients’ demographic characteristics.

	Study 303			Study 304		
	PBO (n = 318)	LEM5 (n = 316)	LEM10 (n = 315)	PBO (n = 208)	LEM5 (n = 266)	LEM10 (n = 269)
Age, y						
Mean (SD)	54.5 (14.0)	54.2 (13.7)	54.8 (13.7)	63.4 (6.4)	63.7 (6.8)	64.2 (6.9)
Median (range)	56.0 (18–83)	55.0 (20–85)	55.0 (18–88)	62 (55–82)	63 (55–88)	64 (55–85)
≥65 [n (%)]	89 (28.0)	87 (27.5)	86 (27.3)	93 (44.7)	118 (44.4)	122 (45.4)

Sex, female [n (%)]	216 (67.9)	209 (66.1)	222 (70.5)	184 (88.5)	229 (86.1)	230 (85.5)

Race [n (%)]						
White	232 (73.0)	222 (70.3)	225 (71.4)	153 (73.6)	199 (74.8)	202 (75.1)
African American	23 (7.2)	27 (8.5)	26 (8.3)	51 (24.5)	63 (23.7)	62 (23.0)
Japanese	54 (17.0)	53 (16.8)	54 (17.1)	1 (0.5)	0 (0.0)	0 (0.0)
Other Asian	5 (1.6)	8 (2.5)	4 (1.3)	1 (0.5)	2 (0.8)	5 (1.9)
Others	4 (1.3)	6 (1.9)	6 (1.9)	2 (1.0)	2 (0.8)	0 (0.0)

BMI, mean (SD), kg/m^2^	27.2 (5.5)	27.3 (6.3)	27.2 (5.6)	27.5 (5.1)	27.4 (4.7)	27.3 (4.6)

Subjective sleep parameter						
sSOL, min [Mean (SD)]	64.0 (45.2)	62.2 (45.7)	65.0 (44.0)	55.9 (37.4)	65.8 (43.5)	60.9 (42.5)
sSE, % [Mean (SD)]	61.3 (17.8)	63.1 (18.2)	62.0 (17.2)	56.1 (17.3)	56.1 (17.1)	54.3 (18.3)
sWASO, min [Mean (SD)]	132.5 (80.2)	132.8 (82.5)	136.8 (87.4)	170.9 (80.7)	166.8 (82.0)	175.4 (83.5)
sTST, min [Mean (SD)]	304.3 (91.5)	315.5 (93.5)	306.9 (88.0)	276.2 (87.6)	275.7 (83.7)	266.1 (92.2)
sQual [Mean (SD)]	3.84 (1.44)	3.94 (1.27)	3.92 (1.35)	3.87 (1.43)	3.78 (1.35)	3.70 (1.31)
Morning alertness [Mean (SD)]	3.94 (1.56)	3.93 (1.35)	3.93 (1.32)	4.15 (1.49)	4.14 (1.49)	3.99 (1.45)

Objective sleep parameter						
LPS, min [Mean (SD)]	―	―	―	43.9 (33.6)	44.9 (36.5)	44.6 (33.0)
SE, % [Mean (SD)]	―	―	―	68.9 (9.6)	68.4 (11.3)	67.9 (10.8)
WASO, min [Mean (SD)]	―	―	―	111.8 (37.2)	113.4 (39.0)	114.8 (40.0)
TST, min [Mean (SD)]	―	―	―	330.7 (46.3)	328.0 (54.2)	325.1 (52.8)
ISI total score [Mean (SD)]	19.0 (3.1)	19.6 (3.3)	19.1 (3.4)	19.4 (3.6)	18.9 (3.5)	19.0 (3.3)
ISI daytime function [Mean (SD)]	11.0 (2.1)	11.4 (2.0)	11.1 (2.2)	11.2 (2.4)	10.9 (2.4)	10.8 (2.3)
FSS total score [Mean (SD)]	35.2 (13.6)	37.4 (12.7)	36.0 (13.0)	37.5 (13.6)	37.5 (13.5)	37.4 (13.1)

BMI, body mass index; FSS, Fatigue Severity Scale; ISI, Insomnia Severity Index; LEM5, lemborexant 5 mg; LEM10, lemborexant 10 mg; LPS, latency to persistent sleep; PBO, placebo; SD, standard deviation; SE, sleep efficiency; SOL, sleep-onset latency; sQual, subjective sleep quality; sSE, subjective sleep efficiency; sSOL, subjective sleep-onset latency; sTST, subjective total sleep time; sWASO, subjective wake after sleep onset; TST, total sleep time; WASO, wake after sleep onset.

### 3.2 Changes in subjective sleep quality (sQual)

**Fig. 1:**
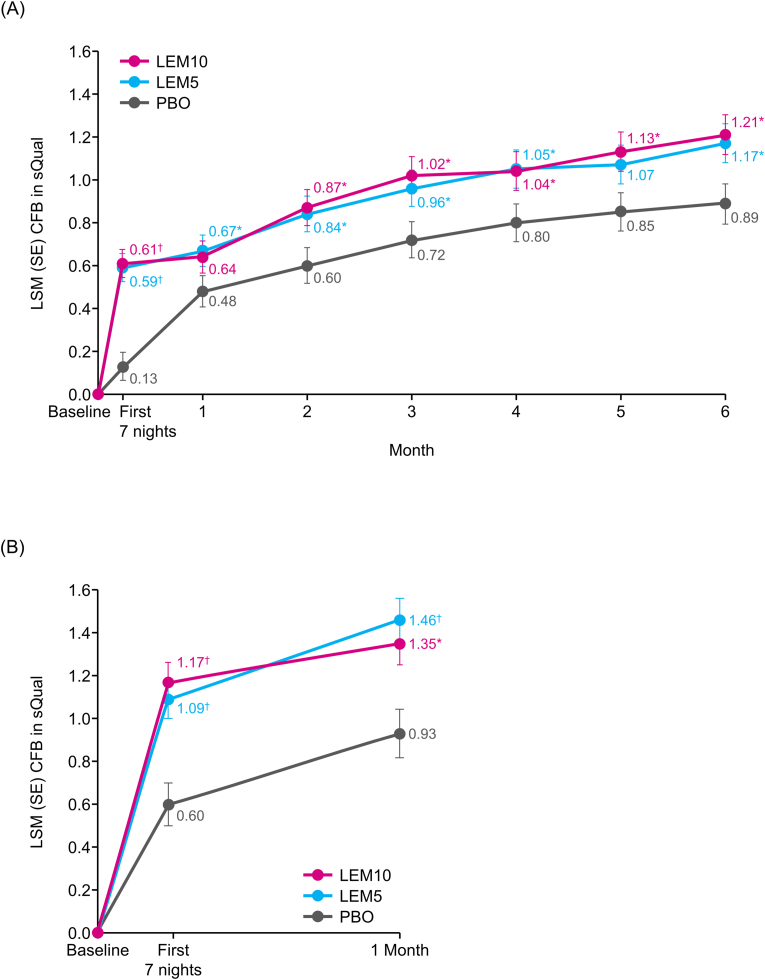
Changes from baseline of sQual in Study 303 (A) and Study 304 (B) ∗p < 0.05, †p < 0.001 versus placebo for change from baseline. CFB, change from baseline; LEM5, lemborexant 5 mg; LEM10, lemborexant 10 mg; LSM, least squares mean; PBO, placebo; SE, standard error; sQual, subjective sleep quality.

In Study 303, both LEM groups had significantly greater increases from baseline in sQual than the placebo group after 7 days of treatment (least square mean change [standard error] from baseline: 0.13 [0.07] for placebo; 0.59 [0.07], p < 0.0001 for LEM5; 0.61 [0.07], p < 0.0001 for LEM10). This significant difference persisted through 6 months, except for Month 1 with LEM10 and Month 5 with LEM5 (Fig. 1A). In Study 304, the increase from baseline in sQual was significantly greater after 7 days (0.60 [0.10] for placebo; 1.09 [0.09], p < 0.0001 for LEM5; 1.17 [0.09], p < 0.0001 for LEM10) and 1 month (0.93 [0.11] for placebo; 1.46 [0.10], p = 0.0002 for LEM5; 1.35 [0.10], p = 0.0029 for LEM10) of treatment in the LEM groups than in the placebo group (Fig. 1B). The changes of sQual from baseline were higher in both LEM groups than in the placebo group across sexes and age groups (<65 years and ≥65 years) (Table S4 and S5).

### 3.3 Responder analysis

**Fig. 2:**
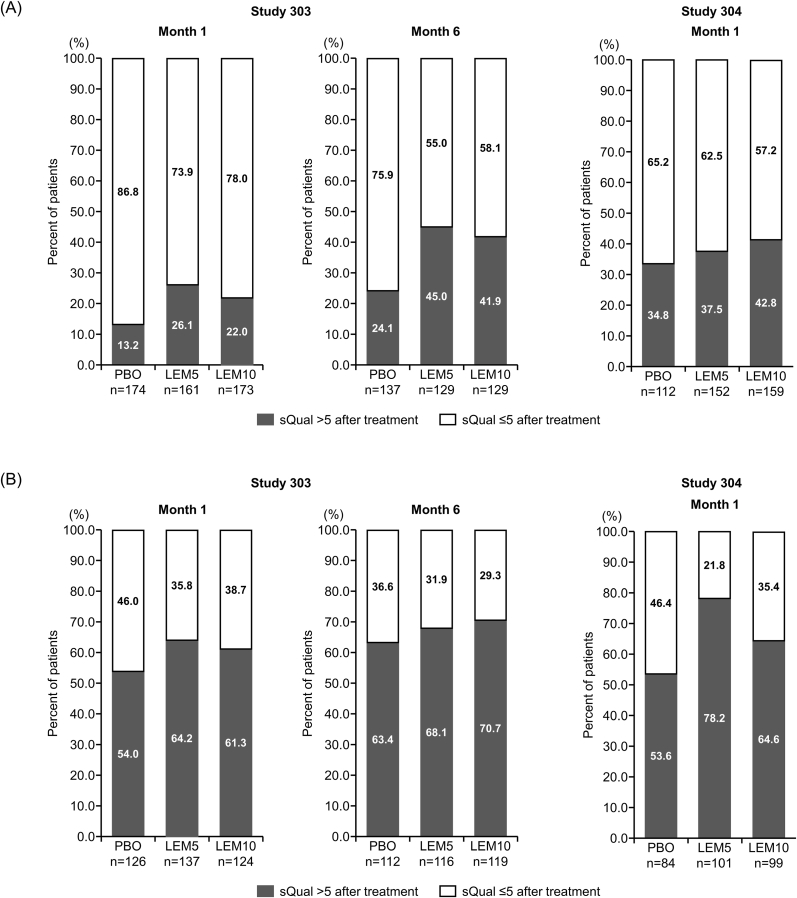
Responder analysis of the proportion of patients in the subgroups with poor baseline sQual (≤4) (A), and in the subgroups with good baseline sQual (>4) at baseline (B). P-values were calculated using the Chi-squared test. LEM5, lemborexant 5 mg; LEM10, lemborexant 10 mg; PBO, placebo; sQual, subjective sleep quality.

In the overall population, the proportion of responders achieving an sQual score >5 was higher in the LEM-treated groups than in the placebo group. In Study 303, these proportions at Month 1 were 30.3% for placebo, 43.6% for LEM5, and 38.4% for LEM10, and at Month 6 were 41.8%, 55.9%, and 54.4%, respectively. Similarly, in Study 304 at Month 1, the corresponding proportions were 42.9% for placebo, 53.8% for LEM5, and 51.2% for LEM10. In Study 303, the proportion of patients who achieved ratings of sQual >5 among those with a baseline rating of sQual ≤4 was higher in the LEM groups than in the placebo group at Month 1 (placebo: 13.2%, LEM5: 26.1%, LEM10: 22.0%) and Month 6 (placebo: 24.1%, LEM5: 45.0%, LEM10: 41.9%). Consistent findings were also observed at Month 1 in Study 304 (placebo: 34.8%, LEM5: 37.5%, LEM10: 42.8%) (Fig. 2A). In those with baseline ratings of sQual >4, more than 60% of patients maintained sQual ratings greater than 5 after 1 to 6 months of treatment with LEM (Fig. 2B).

### 3.4 Correlations between ratings of subjective sleep quality and sleep parameters or subjective morning alertness

All subjective and objective (later assessed only in Study 304) sleep parameters and subjective morning alertness were improved by LEM compared with placebo throughout the study period in both previously described trials (Table S1) [[27], [28], [29], [30], [31]]. The changes in all measures were significantly correlated with the changes in sQual ratings in both studies at Month 1 and in Study 303 at Month 6, except for LPS in Study 304 (p < 0.05) (Table 3). Of the parameters analyzed, only the change in subjective morning alertness had a strong positive correlation with the change in sQual ratings at 1 month in both studies (correlation coefficients: Study 303, LEM5 0.81, LEM10 0.89; Study 304, LEM5 0.78, LEM10 0.75) and at 6 months in Study 303 (LEM5 0.81, LEM10 0.84). Moderate negative correlations of changes in sQual ratings were found with changes in sSOL for LEM5 at one month in Study 303 (−0.40) and with sWASO for both LEM groups at one month in both studies (Study 303, LEM5 −0.51, LEM10 −0.45; Study 304, LEM5 −0.44, LEM10 −0.50) and six months in Study 303 (LEM5 −0.42, LEM10 −0.48). In addition, there were moderate positive correlations between changes in sQual and sTST for both LEM groups at one month in both studies (Study 303, LEM5 0.54, LEM10 0.58; Study 304, LEM5 0.53, LEM10 0.55) and six months in Study 303 (LEM5 0.46, LEM10 0.50). However, the changes in objective sleep parameters in Study 304 had a negligible to weak correlation with the change in sQual ratings at one month. WASO had a stronger correlation than sleep latency for both LEM groups in both studies (Table 3).

**Table 3:** Correlation coefficients between sQual and sleep parameters or morning alertness.

	Study 303				Study 304	
	Month 1		Month 6		Month 1	
	LEM5	LEM10	LEM5	LEM10	LEM5	LEM10
Subjective parameter						
sSOL	−0.40∗∗	−0.24∗∗	−0.38∗∗	−0.35∗∗	−0.28∗∗	−0.30∗∗
sWASO	−0.51∗∗	−0.45∗∗	−0.42∗∗	−0.48∗∗	−0.44∗∗	−0.50∗∗
sTST	0.54∗∗	0.58∗∗	0.46∗∗	0.50∗∗	0.53∗∗	0.55∗∗
Morning alertness	0.81∗∗	0.89∗∗	0.81∗∗	0.84∗∗	0.78∗∗	0.75∗∗

Objective parameter						
LPS					−0.06	−0.06
WASO					−0.35∗∗	−0.14∗
TST					0.34∗∗	0.16∗

∗p < 0.05, ∗∗p < 0.0001.

LEM5, lemborexant 5 mg; LEM10, lemborexant 10 mg; LPS, latency to persistent sleep; sQual, subjective sleep quality; sSOL, subjective sleep-onset latency; sTST, subjective total sleep time; sWASO, subjective wake after sleep onset; TST, total sleep time; WASO, wake after sleep onset.

### 3.5 Stepwise regression analysis

Stepwise regression analysis was conducted with sQual as the dependent variable and sSOL, sWASO, sTST, and subjective morning alertness as the independent variables. The independent variables were sequentially removed until moderate multicollinearity and significance were observed across all final models (variance inflation factor = 1.2–1.6, p-value <0.05) (Table 4). The analysis indicated that changes in subjective morning alertness ratings were the most strongly associated with changes in sQual in Study 303 at Month 1 and Month 6, as well as in Study 304 at Month 1. However, other subjective sleep parameters incorporated in subjective sleep parameters other than morning alertness differed by study or time point. Specifically, in Study 303 at Month 1, sTST and sSOL were included in the final model. Conversely, in Study 303 at Month 6, sWASO and sSOL were included. In Study 304 at Month 1, only sTST was retained in the final model.

**Table 4:** Stepwise regression analysis.

	Full model				Final model				
	Estimate	Standardized coefficient	P-value	VIF	Estimate	Standardized coefficient	P-value	VIF	R^2^
Study 303 at Month 1									
Intercept	0.084		0.0012		0.085		0.0011		0.75
sSOL	−0.002	−0.055	0.0104	1.6	−0.002	−0.044	0.0298	1.4	
sWASO	−0.001	−0.045	0.1132	2.8	removed				
sTST	0.003	0.154	<0.0001	3.8	0.004	0.194	<0.0001	1.6	
Morning alertness	0.769	0.737	<0.0001	1.3	0.770	0.739	<0.0001	1.3	

Study 303 at Month 6									
Intercept	0.140		0.0015		0.150		0.0004		0.71
sSOL	−0.005	−0.107	<0.0001	1.5	−0.006	−0.129	<0.0001	1.2	
sWASO	−0.002	−0.095	0.0046	2.6	−0.003	−0.130	<0.0001	1.2	
sTST	0.001	0.062	0.1064	3.4	removed				
Morning alertness	0.761	0.726	<0.0001	1.3	0.776	0.736	<0.0001	1.2	

Study 304 at Month 1									
Intercept	0.171		0.0003		0.170		0.0002		0.71
sSOL	0.000	0.006	0.8200	1.7	removed				
sWASO	−0.001	−0.045	0.3500	5.4	removed				
sTST	0.004	0.196	0.0004	7.1	0.005	0.233	<0.0001	1.4	
Morning alertness	0.767	0.694	<0.001	1.4	0.767	0.694	<0.0001	1.4	

sSOL, subjective sleep-onset latency; sTST, subjective total sleep time; sWASO, subjective wake after sleep onset; VIF, variance inflation factor.

### 3.6 Analysis of correlations with ISI/FSS

As previously reported, the ISI total score and ISI daytime function score (sum of items 4–7) from baseline were decreased from baseline more in the LEM groups than in the placebo group, with significant differences at Month 1 in both studies and Month 6 in Study 303 (Table S1) [36,37]. The ratings of FSS showed greater decreases from baseline in the LEM groups than in the placebo group in both studies, but a significant difference compared with placebo was observed only in Study 303 [31]; the details are mentioned in the previous reports [27,31,36,37]. Changes in the ISI daytime function score and the FSS score were weakly to moderately negatively correlated with changes in sQual ratings at one month in both studies and six months in Study 303 (correlation coefficients for ISI daytime function and FSS, respectively: Study 303 at 1 month, LEM5 −0.36 and −0.21, LEM10 −0.37 and −0.18; Study 303 at 6 months, LEM5 −0.54 and −0.35, LEM10 −0.42 and −0.21; Study 304 at 1 month, LEM5 −0.38 and −0.20, LEM10 −0.42 and −0.28) (Table 5).

**Table 5:** Correlation coefficients between sQual and daytime function or fatigue.

	Study 303				Study 304	
	Month 1		Month 6		Month 1	
	LEM5	LEM10	LEM5	LEM10	LEM5	LEM10
Daytime function	−0.36∗∗	−0.37∗∗	−0.54∗∗	−0.42∗∗	−0.38∗∗	−0.42∗∗
Fatigue	−0.21∗	−0.18∗	−0.35∗∗	−0.21∗	−0.20∗	−0.28∗∗

∗p < 0.05, ∗∗p < 0.0001.

LEM5, lemborexant 5 mg; LEM10, lemborexant 10 mg; sQual, subjective sleep quality.

## 4 Discussion

Subjective sleep quality has been reported to be independently associated with QOL and health-related outcomes [17], reiterating the importance of evaluating subjective sleep quality. This post hoc analysis showed that sQual ratings improved significantly more in the LEM groups than in the placebo group. On correlation analysis, changes in subjective sleep parameters were more associated with changes in sQual ratings than in objective ones. Subjective morning alertness showed the strongest correlation with sQual in both correlation and stepwise regression analyses. In addition, sTST and sWASO had moderate associations with sQual, positively and negatively, respectively. Moreover, the changes in sQual were negatively correlated with the ISI daytime function score and fatigue.

### 4.1 Effects of pharmacological treatment on subjective sleep quality

There is limited evidence that successful pharmacological treatment for insomnia is associated with improvement in ratings of sQual. Improvements in sQual were seen from 2 days to 4 weeks of treatment in studies of zolpidem and eszopiclone and through 3 months of treatment in a 3-month, randomized, clinical trial of suvorexant [[21], [22], [23]]. In a 3-month, randomized, clinical trial of daridorexant, the sQual ratings were numerically higher at 1 and 3 months after daridorexant administration compared with placebo, but they were not tested statistically [24]. The present study showed improvements in sQual ratings across the first 7 days through at least 6 months of treatment with LEM. These results were consistent with the recent network meta-analysis in which LEM, as well as eszopiclone, demonstrated the best profile in subjective sleep quality or satisfaction for both short- and long-term efficacy among insomnia medications [27,28,38]. In contrast, the between-group differences between LEM and placebo in the change from baseline in sQual ranged from 0.3 to 0.5. Responder analyses, however, suggested a shift in evaluations from neutral ratings toward ratings of good quality. In this analysis, a greater proportion of patients in the LEM groups shifted to an sQual score >5 compared with the placebo group. Accordingly, the between-group differences in mean sQual change may be explained by differences in the proportions of patients achieving higher sQual ratings. This interpretation is supported by the consistency between the between-group differences in responder proportions based on sQual and those based on sWASO [39], which showed a moderate correlation with sQual in the present study.

The sQual ratings were higher in the LEM groups than in the placebo group in all subgroups. This can be expected, since there are no significant differences in the pharmacokinetics of LEM by sex or age [40]. There were no statistically significant differences due to the small number of patients; therefore, further study will be needed.

### 4.2 Relationships between improved subjective sleep quality and other parameters

Correlation analysis showed that the change in sQual had moderate correlations with the changes in several subjective sleep parameters, suggesting that the change in sQual reflected an overall improvement in the subjective sleep state. In contrast, the change in sQual ratings correlated weakly with the changes in objective sleep parameters. A previous study using randomized, clinical trials’ data of suvorexant or gaboxadol including placebo reported weak correlations between subjective sleep quality and objective sleep parameters when considering repeated treatment [41]. These results suggest that subjective sleep quality cannot be explained solely by objective sleep parameters; thus, it may reflect a combination of factors, including those not assessed by PSG. Even if people objectively sleep a similar length of time, how they subjectively evaluate that sleep differs from person to person. This might be associated with individual differences in sleep perception, with individual differences in required sleep time, and/or other objective features of sleep that have yet to be evaluated. Thus, ratings of subjective sleep quality may be more representative of healthy sleep than any of these features alone. Additionally, subjective and objective sleep measures do not completely align even in healthy individuals, and the discrepancy is greater in patients with insomnia [42]. This discrepancy may contribute to the differing correlations between subjective and objective sleep parameters and sQual. Of the objective sleep parameters, the correlation coefficients of changes in WASO and TST were higher than that of change in LPS. This result is consistent with the previous report that identified the relationships between home-based electroencephalography and subjective sleep quality [43].

Spearman's correlation analysis and stepwise regression analysis showed that the subjective morning alertness most strongly correlated with sQual, suggesting that subjective morning alertness significantly contributes to the sQual rating of the previous night. The effects of LEM on sleep with few morning residual effects [30] may have led to higher ratings of subjective morning alertness, as well as better sQual ratings. The previous quantitative choice-based study targeting good and poor sleepers to conceptualize sleep quality judgment also demonstrated that morning alertness is an important factor defining subjective sleep quality [44]. However, among the 17 determinants examined, the top three determinants of subjective sleep quality were total sleep time, feeling refreshed upon waking, and next-day mood in the previous study [44], whereas morning alertness emerged as the strongest correlate of subjective sleep quality in our study. Common method biases [45]—arising from the similarity between the evaluations of subjective sleep quality and morning alertness, both assessed using single-item scales at the same time point—may have contributed to the high correlation observed, potentially resulting in the strongest association in our study. Few residual effects of LEM may result from its pharmacodynamic properties, as its plasma concentration decreases by approximately 70% within 8 h after administration [46]. The recent report showed that sleep inertia was associated with sleep duration or insomnia [47]; therefore, maintaining consolidated sleep and ensuring sufficient sleep time would improve ratings of morning alertness, which in turn would improve sQual ratings. Therefore, to improve sQual, insomnia treatment should also aim to improve sleep continuity.

Changes in sTST and sWASO showed moderate correlations with changes in sQual positively and negatively, respectively, on Spearman's correlation analyses. This finding further supports the argument presented earlier. In addition, LEM has been shown to improve these sleep parameters in clinical trials [27,29,30,48], and these improvements likely contributed to higher sQual ratings.

Finally, sQual ratings had a moderate negative correlation with the ISI daytime function score, suggesting that the effect of LEM on subjective sleep quality may be associated with better daytime function in insomnia patients, aligning with the aforementioned higher ratings of subjective morning alertness.

### 4.3 Strengths and limitations

The strengths of this study include the leveraging of data from large-scale, long-term, double-blind, placebo-controlled, randomized, global, phase 3 studies. Most patients in both studies had a history of at least one medical condition, with postmenopausal status or hypertension being the most common, which is also representative of clinical practice. Allowing the inclusion of a large number of patients with such comorbidities increases the external validity beyond these clinical trials.

There are, however, some limitations. First, causal relationships between sQual ratings and other parameters are unclear, since the analysis investigated only the correlations. Second, potential confounders—such as baseline insomnia severity, comorbid conditions (for example, depression or hypertension) [49,50], psychological status, and history of medication use—may influence changes in sQual; therefore, further analyses, including subgroup analysis, will be necessary. Third, although the PSQI, which consists of multi-item questionnaires for assessing, is the most widely used method to evaluate sleep quality from multiple dimensions [51], we evaluated sleep quality by using a single-item questionnaire in the present study. Therefore, it is difficult to compare the current finding and previous one. However, the single-item questionnaire could more directly reflect subjective feeling of sleep quality per se, and is easy to administer and reduce respondent burden. Fourth, the relationship between objective sleep parameters and sQual was only partially elucidated because these parameters were included exclusively in Study 304, which lasted for one month and enrolled elderly patients, most of whom were female. Fifth, since this study was based on data on LEM, the generalizability of the findings to other treatments with different mechanisms of action should be evaluated in future research. Finally, the fact that this post hoc analysis had not been pre-planned when the studies were conducted is a limitation.

### 4.4 Conclusion

The present analysis of two Phase 3 clinical studies of LEM found that LEM improved sQual ratings more than placebo. Morning alertness was the most strongly associated with sQual of the subjective and objective sleep parameters. This finding suggests that feeling greater alertness in the morning may lead to better subjective ratings of sleep quality and daytime function. Enhancing morning alertness may result from extended sTST or reduced sWASO. Therefore, insomnia treatment should also focus on promoting sleep continuity to improve subjective sleep quality.

## CRediT authorship contribution statement

**Masahiro Suzuki:** Writing – review & editing, Writing – original draft, Visualization, Supervision, Methodology. **Takuya Yoshiike:** Writing – review & editing, Writing – original draft, Visualization, Supervision, Methodology, Conceptualization. **Atul Khullar:** Writing – review & editing, Writing – original draft, Visualization, Supervision, Methodology. **Yuki Kogo:** Writing – review & editing, Writing – original draft, Visualization, Methodology, Conceptualization. **Kanako Inabe:** Writing – review & editing, Writing – original draft, Visualization, Methodology, Conceptualization. **Michinori Koebis:** Writing – review & editing, Writing – original draft, Visualization, Methodology, Conceptualization. **Margaret Moline:** Writing – review & editing, Writing – original draft, Visualization, Methodology, Conceptualization. **Jocelyn Y. Cheng:** Writing – review & editing, Writing – original draft, Visualization, Methodology, Conceptualization. **Dinesh Kumar:** Writing – review & editing, Writing – original draft, Visualization, Methodology, Formal analysis, Data curation, Conceptualization. **Kate Pinner:** Writing – review & editing, Writing – original draft, Visualization, Methodology, Formal analysis, Data curation, Conceptualization. **Kenichi Kuriyama:** Writing – review & editing, Writing – original draft, Visualization, Supervision, Methodology, Conceptualization.

## Funding

This study was financially supported by Eisai Inc., Nutley, NJ, USA. Eisai is the owner and manufacturer of LEM.

## Declaration of competing interest

The authors declare the following financial interests/personal relationships which may be considered as potential competing interests: Masahiro Suzuki and Takuya Yoshiike reports financial support was provided by Eisai Co Ltd. Kenichi Kuriyama reports financial support was provided by Eisai Co Ltd. Masahiro Suzuki reports a relationship with EA Pharma Co Ltd that includes: funding grants and speaking and lecture fees. Masahiro Suzuki reports a relationship with Eisai Co Ltd that includes: funding grants, paid expert testimony, and speaking and lecture fees. Masahiro Suzuki reports a relationship with Kao Corporation that includes: paid expert testimony and speaking and lecture fees. Masahiro Suzuki reports a relationship with Kyowa Pharmaceutical Industry Co Ltd that includes: speaking and lecture fees. Masahiro Suzuki reports a relationship with Meiji Seika Pharma Co Ltd that includes: funding grants and speaking and lecture fees. Masahiro Suzuki reports a relationship with Mochida Pharmaceutical Co Ltd that includes: funding grants, paid expert testimony, and speaking and lecture fees. Masahiro Suzuki reports a relationship with MSD KK that includes: speaking and lecture fees. Masahiro Suzuki reports a relationship with Otsuka Pharmaceutical Co Ltd that includes: funding grants and speaking and lecture fees. Masahiro Suzuki reports a relationship with Shionogi and Co Ltd that includes: funding grants and speaking and lecture fees. Masahiro Suzuki reports a relationship with Sumitomo Pharma Co Ltd that includes: funding grants and speaking and lecture fees. Masahiro Suzuki reports a relationship with Takeda Pharmaceutical Company Limited that includes: speaking and lecture fees. Masahiro Suzuki reports a relationship with Viatris Inc that includes: speaking and lecture fees. Masahiro Suzuki reports a relationship with Yoshitomiyakuhin Corporation that includes: speaking and lecture fees. Masahiro Suzuki reports a relationship with Jazz Pharmaceuticals Inc that includes: paid expert testimony. Takuya Yoshiike reports a relationship with Eisai Co Ltd that includes: consulting or advisory and speaking and lecture fees. Takuya Yoshiike reports a relationship with MSD KK that includes: consulting or advisory and speaking and lecture fees. Takuya Yoshiike reports a relationship with Japan Society for the Promotion of Science that includes: funding grants. Takuya Yoshiike reports a relationship with Viatris Inc that includes: speaking and lecture fees. Takuya Yoshiike reports a relationship with Mitsubishi Tanabe Pharma Corporation that includes: speaking and lecture fees. Atul Khullar reports a relationship with Eisai Inc that includes: board membership, consulting or advisory, and speaking and lecture fees. Atul Khullar reports a relationship with Idorsia Pharmaceuticals Canada Ltd that includes: board membership, consulting or advisory, and speaking and lecture fees. Atul Khullar reports a relationship with AbbVie Inc that includes: board membership, consulting or advisory, and speaking and lecture fees. Atul Khullar reports a relationship with Otsuka Pharmaceutical Co Ltd that includes: board membership, consulting or advisory, and speaking and lecture fees. Atul Khullar reports a relationship with Elvium that includes: board membership, consulting or advisory, and speaking and lecture fees. Atul Khullar reports a relationship with Takeda Pharmaceutical Company Limited that includes: board membership, consulting or advisory, and speaking and lecture fees. Atul Khullar reports a relationship with Jazz Pharmaceuticals Inc that includes: board membership, consulting or advisory, and speaking and lecture fees. Atul Khullar reports a relationship with Lundbeck Japan KK that includes: board membership, consulting or advisory, and speaking and lecture fees. Atul Khullar reports a relationship with Bausch Health Companies Inc that includes: board membership, consulting or advisory, and speaking and lecture fees. Yuki Kogo, Kanako Inabe, and Michinori Koebis reports a relationship with Eisai Co Ltd that includes: employment. Margaret Moline, Jocelyn Y. Cheng, and Dinesh Kumar reports a relationship with Eisai Inc that includes: employment. Kate Pinner reports a relationship with Eisai Ltd that includes: employment. Kenichi Kuriyama reports a relationship with Eisai Co Ltd that includes: consulting or advisory and funding grants. Kenichi Kuriyama reports a relationship with Otsuka Pharmaceutical Co Ltd that includes: funding grants. Kenichi Kuriyama reports a relationship with MSD KK that includes: funding grants. Kenichi Kuriyama reports a relationship with Takeda Pharmaceutical Company Limited that includes: funding grants. Kenichi Kuriyama reports a relationship with Mitsubishi Tanabe Pharma Corporation that includes: funding grants. Kenichi Kuriyama reports a relationship with Shionogi and Co Ltd that includes: consulting or advisory and funding grants. Kenichi Kuriyama reports a relationship with Sumitomo Pharma Co Ltd that includes: funding grants. Kenichi Kuriyama reports a relationship with Daiichi Sankyo Co Ltd that includes: funding grants. Kenichi Kuriyama reports a relationship with Accuris Healthcare LLP that includes: funding grants. Kenichi Kuriyama reports a relationship with Taisho Pharmaceutical Co Ltd that includes: consulting or advisory. Kenichi Kuriyama reports a relationship with Nxera Pharma Japan Co Ltd that includes: consulting or advisory. Kenichi Kuriyama reports a relationship with Nobelpharma Co Ltd that includes: consulting or advisory. Kenichi Kuriyama reports a relationship with Sleep Healthcare Association that includes: consulting or advisory. If there are other authors, they declare that they have no known competing financial interests or personal relationships that could have appeared to influence the work reported in this paper.

## Data Availability

The datasets generated during and/or analyzed during the current study are available from the corresponding author upon reasonable request.

## Glossary

**DSM**: Diagnostic & Statistical Manual of Mental Disorders
**FSS**: Fatigue Severity Scale
**ISI**: Insomnia Severity Index
**LEM**: Lemborexant
**LPS**: Latency to persistent sleep
**MMRM**: Mixed-model repeated measures
**PBO**: Placebo
**PSG**: Polysomnography
**QOL**: Quality of life
**SD**: Standard deviation
**SE**: Sleep efficiency
**sSE**: Subjective sleep efficiency
**sSOL**: Subjective sleep onset latency
**sTST**: Subjective total sleep time
**sWASO**: Subjective wake after sleep onset
**TST**: Total sleep time
**sQual**: Subjective sleep quality
**WASO**: Wake after sleep onset
**ZOL-ER**: Zolpidem extended-release

## References

[1] Cappuccio F.P., D'Elia L., Strazzullo P., Miller M.A. (2010). Sleep duration and all-cause mortality: a systematic review and meta-analysis of prospective studies. Sleep.

[2] da Silva A.A., de Mello R.G., Schaan C.W., Fuchs F.D., Redline S., Fuchs S.C. (2016). Sleep duration and mortality in the elderly: a systematic review with meta-analysis. BMJ Open.

[3] Yin J., Jin X., Shan Z. (2017). Relationship of sleep duration with all-cause mortality and cardiovascular events: a systematic review and dose-response meta-analysis of prospective cohort studies. J Am Heart Assoc.

[4] Zhuang S., Huang S., Huang Z. (2023). Prospective study of sleep duration, snoring and risk of heart failure. Heart.

[5] Lao X.Q., Liu X., Deng H.B. (2018). Sleep quality, sleep duration, and the risk of coronary heart disease: a prospective cohort study with 60,586 adults. J Clin Sleep Med.

[6] Wang D., Li W., Cui X. (2016). Sleep duration and risk of coronary heart disease: a systematic review and meta-analysis of prospective cohort studies. Int J Cardiol.

[7] Shan Z., Ma H., Xie M. (2015). Sleep duration and risk of type 2 diabetes: a meta-analysis of prospective studies. Diabetes Care.

[8] Martin J.L., Fiorentino L., Jouldjian S., Mitchell M., Josephson K.R., Alessi C.A. (2011). Poor self-reported sleep quality predicts mortality within one year of inpatient post-acute rehabilitation among older adults. Sleep.

[9] Andrechuk C.R., Ceolim M.F. (2016). Sleep quality and adverse outcomes for patients with acute myocardial infarction. J Clin Nurs.

[10] Zhang L., Li T., Chen L. (2021). Association of sleep quality before and after SARS-CoV-2 infection with clinical outcomes in hospitalized patients with COVID-19 in China. EXCLI J.

[11] Lee K.S., Lennie T.A., Heo S., Song E.K., Moser D.K. (2016). Prognostic importance of sleep quality in patients with heart failure. Am J Crit Care.

[12] Kaplan K.A., Hirshman J., Hernandez B. (2017). Osteoporotic Fractures in Men (MrOS), Study of osteoporotic Fractures SOF Research Groups. When a gold standard isn't so golden: lack of prediction of subjective sleep quality from sleep polysomnography. Biol Psychol.

[13] Landry G.J., Best J.R., Liu-Ambrose T. (2015). Measuring sleep quality in older adults: a comparison using subjective and objective methods. Front Aging Neurosci.

[14] Maglione J.E., Ancoli-Israel S., Peters K.W. (2014). Study of Osteoporotic Fractures Research Group. Subjective and objective sleep disturbance and longitudinal risk of depression in a cohort of older women. Sleep.

[15] Bernstein J.P.K., DeVito A., Calamia M. (2019). Subjectively and objectively measured sleep predict differing aspects of cognitive functioning in adults. Arch Clin Neuropsychol.

[16] Aziz M., Ali S.S., Das S. (2017). Association of subjective and objective sleep duration as well as sleep quality with non-invasive markers of sub-clinical cardiovascular disease (CVD): a systematic review. J Atherosclerosis Thromb.

[17] Matsui K., Yoshiike T., Nagao K. (2021). Association of subjective quality and quantity of sleep with quality of life among a general population. Int J Environ Res Publ Health.

[18] Künstler E.C.S., Bublak P., Finke K. (2023). The relationship between cognitive impairments and sleep quality measures in persistent insomnia disorder. Nat Sci Sleep.

[19] Hall M., Buysse D.J., Nowell P.D. (2000). Symptoms of stress and depression as correlates of sleep in primary insomnia. Psychosom Med.

[20] Okajima I., Komada Y., Inoue Y. (2011). A meta-analysis on the treatment effectiveness of cognitive behavioral therapy for primary insomnia. Sleep Biol Rhythm.

[21] Uchimura N., Kamijo A., Kuwahara H. (2012). A randomized placebo-controlled polysomnographic study of eszopiclone in Japanese patients with primary insomnia. Sleep Med.

[22] Xiang T., Cai Y., Hong Z., Pan J. (2021). Efficacy and safety of Zolpidem in the treatment of insomnia disorder for one month: a meta-analysis of a randomized controlled trial. Sleep Med.

[23] Herring W.J., Connor K.M., Snyder E. (2016). Suvorexant in patients with insomnia: pooled analyses of three-month data from phase-3 randomized controlled clinical trials. J Clin Sleep Med.

[24] Mignot E., Mayleben D., Fietze I. (2022). Safety and efficacy of daridorexant in patients with insomnia disorder: results from two multicentre, randomised, double-blind, placebo-controlled, phase 3 trials. Lancet Neurol.

[25] Libman E., Fichten C., Creti L. (2016). Refreshing sleep and sleep continuity determine perceived sleep quality. Sleep Disord.

[26] Vermeeren A. (2004). Residual effects of hypnotics: epidemiology and clinical implications. CNS Drugs.

[27] Rosenberg R., Murphy P., Zammit G. (2019). Comparison of lemborexant with placebo and zolpidem tartrate extended release for the treatment of older adults with insomnia disorder: a phase 3 randomized clinical trial. JAMA Netw Open.

[28] Kärppä M., Yardley J., Pinner K. (2020). Long-term efficacy and tolerability of lemborexant compared with placebo in adults with insomnia disorder: results from the phase 3 randomized clinical trial SUNRISE 2. Sleep.

[29] Yardley J., Kärppä M., Inoue Y. (2021). Long-term effectiveness and safety of lemborexant in adults with insomnia disorder: results from a phase 3 randomized clinical trial. Sleep Med.

[30] Moline M., Zammit G., Yardley J. (2021). Lack of residual morning effects of lemborexant treatment for insomnia: summary of findings across 9 clinical trials. Postgrad Med J.

[31] Chepke C., Jain R., Rosenberg R. (2022). Improvement in fatigue and sleep measures with the dual orexin receptor antagonist lemborexant in adults with insomnia disorder. Postgrad Med J.

[32] Yardley J., Inoue Y., Pinner K. (2023). Efficacy and safety of lemborexant in subjects previously treated with placebo for 6 months in a randomized phase 3 study. Sleep Med.

[33] Kushida C.A., Zammit G.K., Cheng J.Y. (2025). Effect of lemborexant on sleep architecture in participants with insomnia disorder and mild obstructive sleep apnea. Sleep Med.

[34] Bastien C.H., Vallières, Morin C.M. (2001). Validation of the Insomnia Severity Index as an outcome measure for insomnia research. Sleep Med.

[35] Schober P., Boer C., Schwarte L.A. (2018). Correlation coefficients: appropriate use and interpretation. Anesth Analg.

[36] Roth T., Rosenberg R., Morin C.M. (2022). Impact of lemborexant treatment on insomnia severity: analyses from a 12-month study of adults with insomnia disorder. Sleep Med.

[37] Chepke C., Cote K.A., Pinner K., Yardley J., Lundwall C., Moline M. (2025). Effect of lemborexant on daytime functioning in adults with insomnia: patient-reported outcomes from a phase 3 clinical trial. Prim Care Companion CNS Disord.

[38] De Crescenzo F., D’Alò G.L., Ostinelli E.G. (2022). Comparative effects of pharmacological interventions for the acute and long-term management of insomnia disorder in adults: a systematic review and network meta-analysis. Lancet.

[39] Citrome L., Juday T., Frech F., Atkins N. (2021). Lemborexant for the treatment of insomnia: direct and indirect comparisons with other hypnotics using number needed to treat, number needed to harm, and likelihood to be helped or harmed. J Clin Psychiatry.

[40] Landry I., Nakai K., Ferry J., Aluri J., Hall N., Lalovic B., Moline M.L. (2021). Pharmacokinetics, pharmacodynamics, and safety of the dual orexin receptor antagonist lemborexant: findings from single-dose and multiple-ascending-dose phase 1 studies in healthy adults. Clin Pharmacol Drug Dev.

[41] Svetnik V., Snyder E.S., Tao P., Roth T., Lines C., Herring W.J. (2020). How well can a large number of polysomnography sleep measures predict subjective sleep quality in insomnia patients?. Sleep Med.

[42] Kay D.B., Buysse D.J., Germain A., Hall M., Monk T.H. (2015). Subjective-objective sleep discrepancy among older adults: associations with insomnia diagnosis and insomnia treatment. J Sleep Res.

[43] Pierson-Bartel R., Ujma P.P. (2024). Objective sleep quality predicts subjective sleep ratings. Sci Rep.

[44] Ramlee F., Sanborn A.N., Tang N.K.Y. (2017). What sways people's judgment of sleep quality? A quantitative choice-making study with good and poor sleepers. Sleep.

[45] Podsakoff P.M., MacKenzie S.B., Lee J.Y., Podsakoff N.P. (2003). Common method biases in behavioral research: a critical review of the literature and recommended remedies. J Appl Psychol.

[46] Inoue Y., Koebis M. (2025). Comprehensive understanding of the treatment of insomnia with lemborexant. Expet Rev Clin Pharmacol.

[47] Kim J.R., Park H.J., Paik S.M. (2026). Morning sleep inertia and its associated factors: findings from a nationwide study. PLoS One.

[48] Laffan A., Caffo B., Swihart B.J., Punjabi N.M. (2010). Utility of sleep stage transitions in assessing sleep continuity. Sleep.

[49] Doi Y., Minowa M., Uchiyama M. (2000). Psychometric assessment of subjective sleep quality using the Japanese version of the Pittsburgh Sleep Quality Index (PSQI-J) in psychiatric disordered and control subjects. Psychiatry Res.

[50] Lo K., Woo B., Wong M., Tam W. (2018). Subjective sleep quality, blood pressure, and hypertension: a meta-analysis. J Clin Hypertens (Greenwich).

[51] Fabbri M., Beracci A., Martoni M., Meneo D., Tonetti L., Natale V. (2021). Measuring subjective sleep quality: a review. Int J Environ Res Publ Health.

