# Supplementary material for: Effects of lemborexant on sleep quality and its association with morning alertness: Post hoc analysis of two phase 3 trials
Source: Sleep Med X. 2026 Mar 5;11:100183. doi: 10.1016/j.sleepx.2026.100183 (PMC13049521; doi:10.1016/j.sleepx.2026.100183)
Supplement: Multimedia component 1 [file mmc1.docx]

**Supplementary materials**

| **Table S1.** | Effects of lemborexant on sleep parameters |
| --- | --- |
| **Table S2.** | Common medical conditions on history in the Safety analysis set of Study 303 |
| **Table S3.** | Common medical conditions on history in the Safety analysis set of Study 304 |
| **Table S4.** | Subgroup analysis of change from baseline in sQual in Study 303 |
| **Table S5.** | Subgroup analysis of change from baseline in sQual in Study 304 |

**Table S1.** Effects of lemborexant on sleep parameters

|  | Study 303 | | |  | | | Study 304 | | |
| --- | --- | --- | --- | --- | --- | --- | --- | --- | --- |
|  | Month 1 | | | Month 6 | | | Month 1 or Days 29/30 | | |
|  | PBO  (N=318) | LEM5  (N=316) | LEM10  (N=315) | PBO  (N=318) | LEM5  (N=316) | LEM10  (N=315) | PBO  (N=208) | LEM5  (N=266) | LEM10  (N=269) |
| sSOL | | | | | | | | | |
| CFB  [Mean (SD)] | −11.5 (32.7) | −19.4 (32.2) | −24.1 (35.2) | −16.6 (35.3) | −29.4 (33.3) | −32.5 (36.0) | −10.7 (36.6) | −29.8 (51.9) | −25.8 (39.1) |
| CFB  [LSGM treatment ratio: Active/Placebo (95% CI)] |  | 0.81 (0.74, 0.89) | 0.77 (0.70, 0.85) |  | 0.73 (0.64, 0.84) | 0.70 (0.61, 0.81) |  | 0.75  (0.67, 0.84) | 0.69  (0.62, 0.77) |
| P-value |  | <0.0001 | <0.0001 |  | <0.0001 | <0.0001 |  | <0.0001 | <0.0001 |
| sWASO | | | | | | | | | |
| CFB  [Mean (SD)] | −19.0 (50.3) | −23.4 (56.3) | −26.8 (57.0) | −32.1 (55.3) | −51.5 (67.3) | −48.1 (68.6) | −36.0 (57.6) | −44.5 (58.1) | −58.0 (72.8) |
| CFB  [LSM treatment difference: Active/Placebo (95% CI)] |  | −5.5  (−13.6, 2.5) | −7.0  (−15.1, 1.1) |  | −17.5  (−27.3, −7.6) | −12.7  (−22.4,  −3.0) |  | −11.5  (−22.4,  −0.6) | −20.6  (−31.5, −9.6) |
| P-value |  | 0.1796 | 0.0898 |  | 0.0005 | 0.0105 |  | 0.0396 | 0.0002 |
| sTST | | | | | | | | | |
| CFB  [Mean (SD)] | 30.7 (70.7) | 39.3 (63.5) | 53.2 (67.9) | 53.5 (74.5) | 76.2 (77.7) | 78.3 (80.7) | 39.0 (66.2) | 62.4 (68.6) | 80.0 (81.2) |
| CFB  [LSM treatment difference: Active/Placebo (95% CI)] |  | 11.8  (1.4, 22.1) | 22.1  (11.8, 32.5) |  | 18.6  (6.1, 31.0) | 22.7  (10.1, 35.2) |  | 23.6  (10.7, 36.5) | 37.8  (24.9, 50.7) |
| P-value |  | 0.0259 | <0.0001 |  | 0.0034 | 0.0004 |  | 0.0003 | <0.0001 |
| Morning alertness | | | | | | | | | |
| CFB  [Mean (SD)] | 0.44 (1.23) | 0.53 (1.17) | 0.55 (1.30) | 0.79 (1.39) | 0.98 (1.46) | 1.05 (1.52) | 0.85 (1.41) | 1.24 (1.50) | 1.18 (1.53) |
| CFB  [LSM treatment difference: Active/Placebo (95% CI)] |  | 0.08  (−0.11, 0.26) | 0.07  (−0.11, 0.26) |  | 0.14  (−0.09, 0.38) | 0.26  (0.03, 0.50) |  | 0.39  (0.13, 0.64) | 0.30 (0.05, 0.56) |
| P-value |  | 0.4120 | 0.4347 |  | 0.2248 | 0.0298 |  | 0.0027 | 0.0182 |
| LPS | | | | | | | | | |
| CFB  [Mean (SD)] |  |  |  |  |  |  | −7.9 (31.9) | −19.5 (33.1) | −21.5 (32.4) |
| CFB  [LSGM treatment ratio: Active/Placebo (95% CI)] |  |  |  |  |  |  |  | 0.77  (0.67, 0.89) | 0.72  (0.63, 0.83) |
| P-value |  |  |  |  |  |  |  | 0.0003 | <0.0001 |
| WASO | | | | | | | | | |
| CFB  [Mean (SD)] |  |  |  |  |  |  | −18.6 (41.9) | −43.9 (39.3) | −46.4 (39.6) |
| CFB  [LSM treatment difference: Active/Placebo (95% CI)] |  |  |  |  |  |  |  | −24.0  (−30.0,  −18.0) | −25.4  (−31.4, −19.3) |
| P-value |  |  |  |  |  |  |  | <0.0001 | <0.0001 |
| TST | | | | | | | | | |
| CFB  [Mean (SD)] |  |  |  |  |  |  | 25.7 (47.6) | 62.0 (46.8) | 67.9 (52.1) |
| CFB  [LSM treatment difference: Active/Placebo (95% CI)] |  |  |  |  |  |  |  | 34.2 (27.0, 41.4) | 38.9 (31.6, 46.1) |
| P-value |  |  |  |  |  |  |  | <0.0001 | <0.0001 |
| ISI total score | | | | | | | | | |
| CFB  [Mean (SD)] | −5.2 (5.2) | −7.1 (5.9) | −7.2 (6.4) | −7.2 (5.8) | −9.9 (6.1) | −9.8 (6.6) | −6.1 (5.5) | −7.8 (5.5) | −7.9 (5.9) |
| CFB  [LSM treatment difference: Active/Placebo  (95% CI)] |  | −1.5 (−2.4, −0.6) | −1.9 (−2.8, −1.0) |  | −2.1 (−3.1, −1.2) | −2.4 (−3.3, −1.4) |  | −1.9 (−2.9, −1.0) | −2.1 (−3.1, −1.1) |
| P-value |  | 0.0013 | <0.0001 |  | <0.0001 | <0.0001 |  | 0.0001 | <0.0001 |
| ISI daytime function (items 4–7) | | | | | | | | | |
| CFB  [Mean (SD)] | −3.1 (3.4) | −4.1 (3.7) | −4.2 (4.0) | −4.3 (3.7) | −6.0 (3.8) | −5.7 (4.0) | −3.9 (3.6) | −4.8 (3.6) | −4.8 (3.7) |
| CFB  [LSM treatment difference: Active/Placebo (95% CI)] |  | −0.7 (−1.3, −0.2) | −0.9 (−1.5, −0.4) |  | −1.3 (−1.9, −0.7) | −1.3 (−1.9, −0.7) |  | −1.1 (−1.7, −0.5) | −1.1 (−1.7, −0.5) |
| P-value |  | 0.0137 | 0.0011 |  | <0.0001 | <0.0001 |  | 0.0006 | 0.0007 |
| FSS | | | | | | | | | |
| CFB  [Mean (SD)] | −3.9 (11.6) | −6.6 (11.8) | −6.4 (13.7) | −6.3 (12.1) | −10.1 (13.6) | −8.9 (14.9) | −6.8 (11.9) | −8.1 (13.4) | −8.0 (14.1) |
| CFB  [LSM treatment difference: Active/Placebo (95% CI)] |  | −1.7 (−3.4, 0.1) | −2.0 (−3.8, −0.3) |  | −2.5 (−4.5, −0.5) | −2.6 (−4.6, −0.5) |  | −1.3 (−3.4, 0.8) | −1.2 (−3.3, 0.9) |
| P-value |  | 0.0670 | 0.0257 |  | 0.0134 | 0.0128 |  | 0.2348 | 0.2745 |

CI, confidence interval; CFB, change from baseline; FSS, Fatigue Severity Scale; ISI, Insomnia Severity Index; LEM5, lemborexant 5 mg; LEM10, lemborexant 10 mg; LPS, latency to persistent sleep; LSGM, least squares geometric mean; LSM, least square mean; PBO, placebo; SD, standard deviation; SE, sleep efficiency; SOL, sleep-onset latency; sQual, subjective sleep quality; sSE, subjective sleep efficiency; sSOL, subjective sleep-onset latency; sTST, subjective total sleep time; sWASO, subjective wake after sleep onset; TST, total sleep time; WASO, wake after sleep onset.

**Table S2**. Common medical conditions on history in the Safety analysis set of Study 303

| N (%) | PBO (n=319) | LEM5  (n=314) | LEM10  (n=314) |
| --- | --- | --- | --- |
| Any medical history | 294 (92.2) | 280 (89.2) | 285 (90.8) |
| Preferred term |  |  |  |
| Depression | 32 (10.0) | 34 (10.8) | 33 (10.5) |
| Headache | 27 (8.5) | 24 (7.6) | 36 (11.5) |
| Hypercholesterolaemia | 33 (10.3) | 40 (12.7) | 32 (10.2) |
| Hypertension | 95 (29.8) | 79 (25.2) | 105 (33.4) |
| Menopause | 57 (17.9) | 50 (15.9) | 54 (17.2) |
| Osteoarthritis | 28 (8.8) | 40 (12.7) | 30 (9.6) |
| Postmenopause | 58 (18.2) | 54 (17.2) | 55 (17.5) |

Medical conditions reported on history in more than 10% of patients in any intervention are listed.

LEM5, lemborexant 5 mg; LEM10, lemborexant 10 mg; PBO, placebo.

**Table S3**. Common medical conditions on history in the Safety analysis set of Study 304

| N (%) | PBO (n=209) | LEM5  (n=266) | LEM10  (n=268) |
| --- | --- | --- | --- |
| Any medical history | 206 (98.6) | 263 (98.9) | 261 (97.4) |
| Preferred term |  |  |  |
| Drug hypersensitivity | 28 (13.4) | 39 (14.7) | 31 (11.6) |
| Female sterilisation | 21 (10.0) | 27 (10.2) | 28 (10.4) |
| Gastroesophageal reflux disease | 32 (15.3) | 27 (10.2) | 39 (14.6) |
| Hypertension | 64 (30.6) | 81 (30.5) | 80 (29.9) |
| Hypothyroidism | 30 (14.4) | 26 (9.8) | 23 (8.6) |
| Hysterectomy | 41 (19.6) | 67 (25.2) | 51 (19.0) |
| Hyperlipidaemia | 22 (10.5) | 28 (10.5) | 34 (12.7) |
| Menopause | 27 (12.9) | 35 (13.2) | 33 (12.3) |
| Osteoarthritis | 24 (11.5) | 43 (16.2) | 30 (11.2) |
| Postmenopause | 111 (53.1) | 137 (51.5) | 137 (51.1) |
| Seasonal allergy | 23 (11.0) | 37 (13.9) | 29 (10.8) |

Medical conditions reported on history in more than 10% of patients in any intervention are listed.

LEM5, lemborexant 5 mg; LEM10, lemborexant 10 mg; PBO, placebo.

**Table S4**. Subgroup analysis of change from baseline in sQual in Study 303

|  | Sex | | | | | | Age (y) | | | | | |
| --- | --- | --- | --- | --- | --- | --- | --- | --- | --- | --- | --- | --- |
|  | Male | | | Female | | | <65 | | | ≥65 | | |
|  | PBO (n=100) | LEM5 (n=106) | LEM10 (n=93) | PBO (n=217) | LEM5 (n=208) | LEM10 (n=219) | PBO (n=229) | LEM5 (n=228) | LEM10 (n=226) | PBO (n=88) | LEM5 (n=86) | LEM10 (n=86) |
| Baseline  [Mean (SD)] | 3.56 (1.34) | 3.93 (1.16) | 3.87 (1.33) | 3.97 (1.46) | 3.94 (1.33) | 3.94 (1.36) | 3.71 (1.48) | 3.84 (1.31) | 3.81 (1.32) | 4.19 (1.26) | 4.21 (1.13) | 4.20 (1.39) |
| CFB at First 7 nights  [LS Mean (SE)] | 0.12 (0.12) | 0.68 (0.11) | 0.58 (0.12) | 0.14 (0.08) | 0.57 (0.08) | 0.63 (0.08) | 0.20 (0.08) | 0.54 (0.08) | 0.66 (0.08) | −0.05 (0.10) | 0.75 (0.11) | 0.49 (0.11) |
| P-value |  | 0.0002 | 0.0027 |  | <0.0001 | <0.0001 |  | 0.0013 | <0.0001 |  | <0.0001 | 0.0001 |
| CFB at Month 1  [LS Mean (SE)] | 0.40 (0.13) | 0.85 (0.12) | 0.57 (0.13) | 0.52 (0.09) | 0.59 (0.09) | 0.68 (0.09) | 0.57 (0.09) | 0.64 (0.09) | 0.77 (0.09) | 0.26 (0.11) | 0.77 (0.12) | 0.33 (0.12) |
| P-value |  | 0.0047 | 0.2946 |  | 0.5945 | 0.1857 |  | 0.5512 | 0.1013 |  | 0.0007 | 0.6396 |
| CFB at Month 2  [LS Mean (SE)] | 0.63 (0.15) | 0.95 (0.14) | 0.76 (0.15) | 0.59 (0.10) | 0.80 (0.10) | 0.93 (0.10) | 0.75 (0.10) | 0.80 (0.10) | 0.96 (0.10) | 0.24 (0.14) | 0.97 (0.14) | 0.62 (0.15) |
| P-value |  | 0.0977 | 0.5335 |  | 0.1414 | 0.0150 |  | 0.7417 | 0.1214 |  | 0.0002 | 0.0516 |
| CFB at Month 3  [LS Mean (SE)] | 0.73 (0.14) | 0.96 (0.14) | 0.94 (0.15) | 0.73 (0.11) | 0.97 (0.11) | 1.05 (0.11) | 0.89 (0.10) | 0.97 (0.10) | 1.05 (0.10) | 0.32 (0.14) | 0.95 (0.14) | 0.90 (0.15) |
| P-value |  | 0.2059 | 0.2818 |  | 0.0898 | 0.0240 |  | 0.5424 | 0.2327 |  | 0.0012 | 0.0032 |
| CFB at Month 4  [LS Mean (SE)] | 0.82 (0.15) | 1.01 (0.15) | 0.99 (0.16) | 0.80 (0.11) | 1.07 (0.11) | 1.07 (0.11) | 0.97 (0.11) | 1.06 (0.11) | 1.09 (0.11) | 0.38 (0.14) | 1.01 (0.14) | 0.92 (0.15) |
| P-value |  | 0.3448 | 0.4067 |  | 0.0691 | 0.0620 |  | 0.5326 | 0.4155 |  | 0.0010 | 0.0052 |
| CFB at Month 5  [LS Mean (SE)] | 0.83 (0.16) | 1.14 (0.15) | 1.04 (0.16) | 0.87 (0.11) | 1.04 (0.11) | 1.17 (0.11) | 1.04 (0.11) | 1.08 (0.11) | 1.23 (0.11) | 0.40 (0.14) | 1.04 (0.15) | 0.87 (0.15) |
| P-value |  | 0.1430 | 0.3317 |  | 0.2574 | 0.0407 |  | 0.7890 | 0.2057 |  | 0.0011 | 0.0170 |
| CFB at Month 6  [LS Mean (SE)] | 0.95 (0.16) | 1.15 (0.15) | 1.06 (0.16) | 0.86 (0.11) | 1.18 (0.11) | 1.27 (0.11) | 1.02 (0.11) | 1.19 (0.11) | 1.29 (0.11) | 0.55 (0.14) | 1.14 (0.14) | 0.98 (0.14) |
| P-value |  | 0.3406 | 0.6219 |  | 0.0376 | 0.0074 |  | 0.2898 | 0.0821 |  | 0.0019 | 0.0238 |

CFB, change from baseline; LEM5, lemborexant 5 mg; LEM10, lemborexant 10 mg; PBO, Placebo; sQual, subjective sleep quality.

**Table S5**. Subgroup analysis of change from baseline in sQual in Study 304

|  | Sex | | | | | | Age (y) | | | | | |
| --- | --- | --- | --- | --- | --- | --- | --- | --- | --- | --- | --- | --- |
|  | Male | | | Female | | | <65 | | | ≥65 | | |
|  | PBO (n=24) | LEM5 (n=37) | LEM10 (n=39) | PBO (n=184) | LEM5 (n=229) | LEM10 (n=230) | PBO (n=115) | LEM5 (n=148) | LEM10 (n=147) | PBO (n=93) | LEM5  (n=118) | LEM10 (n=122) |
| Baseline  [Mean (SD)] | 3.72 (1.12) | 3.38 (1.30) | 3.61 (1.33) | 3.89 (1.47) | 3.85 (1.35) | 3.71 (1.31) | 3.70 (1.46) | 3.79 (1.30) | 3.69 (1.35) | 4.08  (1.37) | 3.77 (1.41) | 3.70 (1.26) |
| CFB at First 7 nights  [LS Mean (SE)] | 0.61 (0.30) | 1.29 (0.24) | 1.44 (0.23) | 0.60 (0.11) | 1.06 (0.10) | 1.13 (0.10) | 0.68 (0.14) | 1.05 (0.12) | 1.21 (0.13) | 0.49 (0.14) | 1.13 (0.13) | 1.11 (0.13) |
| P-value |  | 0.0725 | 0.0240 |  | 0.0004 | <0.0001 |  | 0.0264 | 0.0013 |  | 0.0003 | 0.0005 |
| CFB at Month 1  [LS Mean (SE)] | 1.03 (0.32) | 1.37 (0.26) | 1.37 (0.25) | 0.92 (0.12) | 1.48 (0.11) | 1.35 (0.11) | 1.04 (0.16) | 1.60 (0.14) | 1.50 (0.14) | 0.79 (0.16) | 1.30 (0.14) | 1.16 (0.14) |
| P-value |  | 0.3915 | 0.3857 |  | 0.0002 | 0.0042 |  | 0.0038 | 0.0158 |  | 0.0131 | 0.0686 |

CFB, change from baseline; LEM5, lemborexant 5 mg; LEM10, lemborexant 10 mg; PBO, placebo; sQual, subjective sleep quality.
